# The impact of novel and traditional food bank approaches on food insecurity: a longitudinal study in Ottawa, Canada

**DOI:** 10.1186/s12889-021-10841-6

**Published:** 2021-04-22

**Authors:** Anita Rizvi, Rania Wasfi, Aganeta Enns, Elizabeth Kristjansson

**Affiliations:** 1 School of Psychology, Faculty of Social Sciences, University of Ottawa, 136 Jean-Jacques Lussier Pvt, Room VNR5015, Vanier Hall, Ottawa, Ontario K1N 6N5 Canada; 2 Centre for Surveillance and Applied Research, Public Health Agency of Canada, Government of Canada, Ottawa, Canada

## Abstract

**Background:**

Food insecurity is strongly associated with poor mental and physical health, especially with chronic diseases. Food banks have become the primary long-term solution to addressing food insecurity. Traditionally, food banks provide assistance in the form of pre-packed hampers based on the food supplies on hand, such that the food items often do not meet the recipients’ cultural, religious or medical requirements. Recently, new approaches have been implemented by food banks, including choice models of food selection, additional onsite programming, and integrating food banks within Community Resource Centres.

**Methods:**

This study examined changes in food security and physical and mental health, at four time points over 18 months at eleven food banks in Ottawa, Ontario, Canada. The participants – people who accessed these food banks – were surveyed using the Household Food Security Survey Module (HFSSM) and the Short-Form Health Survey Version 2 (SF-12). Statistical analyses included: pairwise paired t-tests between the mean perceived physical and mental health scores across the four waves of data collection, and longitudinal mixed effects regression models to understand how food security changed over time.

**Results:**

The majority of people who were food insecure at baseline remained food insecure at the 18-month follow-up, although there was a small downward trend in the proportion of people in the severely food insecure category. Conversely, there was a small but significant increase in the mean perceived mental health score at the 18-month follow-up compared to baseline. We found significant reductions in food insecurity for people who accessed food banks that offered a Choice model of food distribution and food banks that were integrated within Community Resource Centres.

**Conclusions:**

Food banks offer some relief of food insecurity but they don’t eliminate the problem. In this study, reductions in food insecurity were associated with food banks that offered a Choice model and those that were integrated within a Community Resource Centre. There was a slight improvement in perceived mental health at the 18-month time point; however, moderately and severely food insecure participants still had much lower perceived mental health than the general population.

## Introduction

Household food insecurity, defined as the inadequate or insecure access to food due to financial constraints, is a growing health problem in Canada that adversely affects mental, physical, and social health, and strains our healthcare system [1, 2]. The magnitude of the problem is alarming considering that in 2017–2018, one in eight households in Canada faced food insecurity, which translates into nearly 4.4 million people, including more than 1.2 million children. The number of people living in food-insecure households in 2017–2018 constitutes the highest rate since national monitoring began in 2007 [2].

Past research has highlighted the many negative health consequences associated with food insecurity [3–5], including a multitude of chronic conditions, such as arthritis, back problems, hypertension, diabetes, and cardiovascular disease [6–9]. Additionally, adults with mobility impairments are inordinately affected by food insecurity [10]. Food insecurity likewise has an enduring effect on children’s wellbeing, with studies linking the exposure to food insecurity at an early age with increased risk of developing asthma, depression, and suicidal ideation in adolescence and early adulthood [11–13].

Food insecurity has been associated with nutritional vulnerability. In Canada, adults in food-insecure households reported lower dietary intake of energy, macronutrients and micronutrients in comparison to their food secure counterparts; adolescents who were food insecure also reported some nutritional deficits [14]. People living in food insecure households reported limited social support and poorer social cohesion in their neighbourhoods [15, 16] compared to food secure households.

Food banks emerged in Canada in the early 1980’s as a short-term measure to ameliorate a surge in food insecurity due to job losses after a downturn in the oil industry and the subsequent economic recession [17]. The number of visits to Canadian food banks has been climbing since then, with 1,084,386 visits reported across the Canadian Food Bank Network in March 2019 [18, 19]. In the absence of comprehensive government policies, food banks have continued to propagate, and these agencies are now the first line of response to the issue of hunger and food insecurity in Canada [20].

With respect to terminology, food banks in Canada serve the functions of both “food pantries” – the local not-for-profit agencies that provide food assistance, in the form of unprepared grocery items, directly to people in need – as well as the central warehouses which are referred to as food banks in the United States, and which distribute food to various types of front-line food programs [21]. It should be noted that the terms “food bank” and “food pantry” may carry different meanings in an international context, for example, the term “food pantry” in the United Kingdom refers to a “membership scheme” which allows members to obtain a limited number of food items, typically redistributed surplus stock from supermarkets, for a nominal weekly fee [22]. Food banks in Canada offer food assistance free of charge, but the frequency of visits is usually limited, typically to once per month, with the goal of providing a few days’ worth of groceries during each visit. In this paper, we use the term “food bank” to refer specifically to local agencies that provide unprepared food items at no cost directly to individuals, with one exception being the Ottawa Food Bank (OFB) organization, which operates a central warehouse facility that serves member agencies in the Ottawa area.

Each food bank that participated in this study serves a specific geographic area of Ottawa. To receive assistance, people do not need referrals from other agencies; however, the food banks may require people to provide documents during their first visit to verify their identity, address, and income. Proof of address may need to be presented at subsequent visits to confirm residence within the area that a food bank serves.

Despite the escalation of food bank use in recent decades, food banks have limited capacity to alleviate the needs of those who seek assistance [23]. Furthermore, although conventional food bank models may be linked with short-term improvement in household food security and health [24], these agencies have a limited capacity to offer food of adequate quality and variety due to their reliance on donations [23]. Furthermore, people report experiencing stigma, embarrassment, frustration and shame when accessing a food bank, because they often receive food that is left over/unsold, high in sugar and fat, and past the best-before date [25, 26].

Change is taking place in the ways that food banks provide food assistance [27]. Contemporary approaches to improving services include increasing the quality and choice of food provisions, establishing safe and welcoming spaces, and providing greater integration with health care and health promotion [20]. Recent studies have examined the potential benefits of Choice models [28–30], in which people visiting food banks can select food items from displays, as in a grocery store, instead of receiving pre-packed hampers. Research is also emerging on food banks which offer an array of services such as nutrition education, life-skills training, and health and social support services, in addition to food assistance [31–35]; however, the existing research documents a significant heterogeneity in the types of supplementary services offered.

Although the number of food banks in Canada has been proliferating for more than four decades, there is a dearth of studies describing and evaluating both traditional strategies as well as the newer, more novel approaches [27, 29, 36, 37]. To help fill this gap, we collaborated with the Ottawa Food Bank (OFB) to plan and carry out this study, which was conducted in collaboration with eleven community food banks within the OFB network.

There is also a gap in the literature regarding the health of people who access food banks, which are a specific sub-population of food insecure people in general. Studies have found that less than one quarter of people in food insecure households in Canada rely on food banks, and that the people who do access food banks are not a representative subset of the food insecure population, having substantially lower incomes and higher rates of receiving social assistance benefits than food insecure people who do not access food banks [38, 39]. We found five quantitative studies that examined the health of people who relied on food banks in Europe and North America [24, 40–43]; however, none of these studies were of a longitudinal nature with participants who accessed food banks on a long-term basis. All the other literature we reviewed on household food insecurity and health relied on data from cross-sectional population surveys.

## Methods

### Study aims

The main aim of this study is to model changes in food insecurity over time and identify their associations with different types of food bank approaches offered in Ottawa. We also report on food bank use and examine change in physical and mental health over the 18-month period.

### Study design

This observational prospective study was conducted from November 2017 until December 2019 and involved repeated surveys of the same cohort of participants over four time points. A baseline survey and three follow-up surveys were conducted at intervals of approximately 6 months, such that there was a total span of approximately 18 months between the baseline survey and the final survey for each participant. (The complete surveys are included in a companion article by Enns [44]).

This study was originally planned to last 2 years, with a fourth follow-up survey at the 24-month time-point; however, due to significant attrition and many surveys from participants being returned incomplete, we chose to end the study after the 18-month follow up, which still provided an adequate sample size to yield statistically meaningful results (details are provided in the Sample Size and Attrition section below). The decision to omit the 24-month time point was also based on receiving feedback from some participants who expressed annoyance over being contacted repeatedly for the follow-up surveys. We determined that an 18-month follow-up would still contribute novel longitudinal evidence as this time period is longer than any previous longitudinal studies of food bank access and trajectories of food insecurity.

### Participants and setting

The participants in this study were people who accessed community food banks in Ottawa, Ontario, Canada. Eleven of twenty-six community food banks within the Ottawa Food Bank (OFB) network were included in this study. The eleven food banks were identified and recruited in collaboration with the OFB, which is the central collection and distribution hub of the network. Partners at the OFB distributed an email to community food bank coordinators within their network that included study information and an invitation to directly contact a member of the University of Ottawa research team (by phone or email) if they were interested in taking part and facilitating data collection at their food bank. The research team member who received correspondence from interested food bank coordinators then invited coordinators to in-person meetings to provide further study information, answer questions, and gather information on food bank operations. Each food bank that participated in this study serves a specific geographic area of Ottawa and provides food to 400 or more people per month.

The participants were recruited in the food bank waiting areas. People were approached and given information about the study, and if they were interested in participating, they were asked to read a consent form. People who were 18 years of age or older and comfortable conversing in English or French were eligible to participate. Those people who provided signed consent were then given several options for completing the initial baseline survey: (i) filling out a paper version, (ii) completing an electronic version on a tablet, (iii) completing the survey in private with a research assistant who would read the questions out, or (iv) completing an online version at home, using the Internet URL provided in a handout.

The six-, twelve- and eighteen-month follow-up surveys were completed over the phone, or by email with a link to access an online version, or by regular mail using a printed paper version which could be returned in a supplied, postage-paid envelope.

As an incentive to join the study, participants in the baseline survey were invited to enter a draw for one of eight $50 grocery store gift cards at the time of consenting to take part in the study. Participants who indicated that they would like to enter the draw were also asked for their preferred contact method and information and were assigned a random ID number. At the end of the baseline data collection periods, IDs were entered into a random number generator to select the eight winners, who received the gift cards by mail. Everyone who participated in the six-month follow-up survey received a $5 grocery store gift card by mail, and everyone who participated in the twelve- and eighteen-month follow-ups received a $10 grocery store gift card by mail for each survey. The amount was increased from $5 to $10 to encourage retention due to the significant attrition which was observed at the six-month follow-up.

### Survey questionnaire design

The survey questionnaire sought to measure the participants’ demographics, duration and frequency of food bank access, level of food insecurity, and self-reported physical health and mental health.

Food security was measured using the Household Food Security Survey Module (HFSSM), an 18-item measure used in national population health surveys in Canada and includes questions on household food security situations over a 12-month period. The HFSSM is based on the Core Food Security Module developed by the United States Department of Agriculture to be a benchmark measure of household food security, which has been used and validated widely in North America [45].

Perceived mental and physical health were measured using the 12-item version of the Short-Form Health Survey Version 2 (SF-12) [46]. The SF-12 is a widely used measure of self-reported health. It has demonstrated good reliability and validity among diverse populations [47]. The SF-12v2 has also been shown to be a valid outcome indicator among marginalized or vulnerable populations [47, 48]. The Physical and Mental Health Composite Scores (PCS and MCS) are continuous variables measured on a scale from 0 to 100, where 0 indicates poor perceived health, and 100 indicates excellent perceived health.

### Statistical analysis

We assessed descriptive statistics to demonstrate demographic characteristics of the study sample. We report the means and standard deviations of participants’ age  and perceived physical and mental health scores at the four waves of data collection. We also summarized the proportions of people with different gender identities, education; monthly income; marital status; whether participants were born in Canada or abroad; their ethnicity; marital status and whether or not they live with dependents.

***To measure change in physical and mental health*** across the four waves of data collection, we performed pairwise paired t-tests between the scores of the physical and mental health of the within-subject factor (i.e., across waves of data collection). *P*-values were adjusted using the Bonferroni multiple testing correction method [49].

***To examine food bank use*** in each of the four waves of data collection, we asked about and reported frequency of use of food banks in the 3 months preceding each survey.

#### Modelling food insecurity

We conducted longitudinal mixed effects regression models [50] to understand how food security changed during the four waves of data collection and to understand their associations with different types of food bank approaches offered in Ottawa. Participants were nested within the four time points of data collection.

### Main outcome measure

We used both categorical and continuous scales as each of them serve a particular purpose in our analysis.

As explained in detail by Carlson et al. [51] and Bickel et al. [45], the Food Security Scale is a continuous linear scale, developed to measure the degree of severity of food insecurity/hunger experienced by a household in terms of a single numerical value on a ten-point scale (i.e., from 1 to 10, where 1 indicates food secure and 10 indicates severely food insecure). We used this scale in the regression models to show the precise change in food security levels, and associations with novel and traditional food bank approaches.

We also decided to show food security as a categorical variable for descriptive purposes, providing a small set of categories, each one representing a meaningful range of severity of food insecurity. Thus, scores were categorized as: 0 = food secure, 1 = marginal food insecurity, 2 = moderate food insecurity, or 3 = severe food insecurity. Categories were created using established criteria for scoring the HFSSM [51]; the cuts offs were developed by Bickel et al. [45].

### Main variables of interest

The main variables of interest (the independent variables / IVs) were the food banking models used in the eleven participating food banks:

1. **Food bank type**: integrated within a Community Resource Centre (CRC IV): a dichotomous variable: 0 = not CRC, 1 = is a CRC.
2. **Choice distribution model** (Choice IV): a dichotomous variable: 0 = Hamper model, 1 = Choice model.
3. **Additional onsite programming** (Programs IV): a dichotomous variable: 0 = no, 1 = yes.

We conducted a Chi-squared test between the CRC and Choice models as well as the CRC and Program models to examine their independence.

Six of the eleven food banks offered additional onsite programming, which included food-related programs such as community kitchens, as well as support for finding employment or affordable housing, or applying for social assistance.

Three of the food banks were situated within Community Resource Centres (CRCs) which provide wraparound services, so that emergency food assistance, community programs, and health and social services were all offered in one place. In comparison, the additional onsite programming model is limited to helping people to find and access such services elsewhere, as the food bank itself is not integrated within a CRC.

Four of the food banks offered food assistance via a choice or ‘grocery shopping’ model, whereas the other seven provided food supplies in the form of a food hamper, with some offering choice of certain items by way of a food options list. In the choice model as referred to in this paper, people are invited to walk around a food display area, typically with a volunteer, and choose food items that they and their family need and want. Choice model food banks may place limits on the number of food items collected per person and per food category.

Food bank characteristics were not mutually exclusive and food banks could possess more than one approach. However, based on the results of our contingency analysis (shown further below) and the aim of this study, each food banking approach was analysed separately.

### Covariates

Individual covariates included in the analyses were: age at baseline, gender, monthly household income, having dependents in the household or not, ethnicity, whether born in Canada or not, married/living with a partner or not, perceived physical health and perceived mental health.

### Sample size and attrition

We used the Generalized Linear Mixed Model Power and Sample Size (GLIMMPSE) software (https://glimmpse.samplesizeshop.org) to estimate the sufficient sample size needed to model food security score, using a multi-level mixed effect model with repeated measures across four waves of data collection. The sufficient sample size estimated to detect a target power of 0.8 with a Type I error rate of 0.05 was 229 participants. Our sample size used in the analysis was 369 participants with 1040 observations across the 4 waves of data collection, which was sufficient to detect a meaningful effect.

Seven hundred and thirty participants were recruited in total at baseline. Participants who did not respond to at least two of the four data collection waves were excluded from the analysis, resulting in a sample of 401 participants at baseline.

Our colleague Enns [44] performed a statistical comparison of all the recruited participants and those who completed the six-month follow-up and did not find any significant differences in their demographic characteristics; i.e., the participants who were excluded at baseline or who did not complete the six-month survey were not significantly different from the follow-up participants, in terms of education, gender, ethnicity, being born in Canada, marital status or having dependents.

In the current study, an attrition analysis was conducted for each of the three follow-ups, to understand whether people who did not participate in some waves of data collection dropped out at random or whether significant differences in sample characteristics existed between people who answered the survey and those who were missing in each wave. No significant differences were found between baseline sample characteristics of the group that answered the survey and those who dropped out in each wave of data collection in terms of age *p*-value (attrition W2 = 0.1073, attrition W3 = 0.2582, attrition W4 = 0.4173), perceived physical health *p*-value (attrition W2 = 0.5273, attrition W3 = 0.5188, attrition W4 = 0.8808), mental health *p*-value (attrition W2 = 0.2912, attrition W3 = 0.3114, attrition W4 = 0.8417), and food security level *p*-value (attrition W2 = 0.7674, attrition W3 = 0.5373, attrition W4 = 0.8808). These results suggest that participants dropped out at random.

In the four waves of data collection for the eighteen-month study, there were: 401 participants who responded with complete data in wave 1; 320 in wave 2; 311 in wave 3; and 271 in wave 4. Some participants skipped a wave, and then returned to answer in a following wave. In total, 189 participants answered all four waves of data collection, 125 participants answered three waves of surveys, and 85 participants answered the two waves of surveys. Across all waves, there were a total of 1303 valid responses, and 301 missing ones.

We imputed missing data only for time-constant variables that were reported by participants in one wave of data collection, but missing in others; for example, if in one wave of data collection a participant did not report their age, gender, education, ethnicity, whether they were born in Canada or not, data was imputed from their answers from another wave. However, for all variables that can change over time – for example food security, income, marital status, perceived mental and physical health – missing data was not imputed.

In longitudinal data analysis using mixed effects regression models, two points in time can be used in the analysis without the need to impute missing data, if the missing data is “missing completely at random”; hence, the analysis provides valid inferences, with no need to impute, delete, or weight [50].

Data preparation, cleaning and analyses were conducted in Stata 13.1 and R Studio 4.0.1.

## Results

### Descriptive statistics

#### Sample characteristics in each wave of data collection

At baseline, 401 participants answered a set of demographic questions. As shown in Table 1, the majority of the sample at baseline were: born in Canada (68.8%), white (53.4%), women (50.9%), not married nor living with a partner (64.3%), with no dependents (52.1%), and had some (i.e., not completed) college education or less (61.8%). Around 79.8% of participants’ household income in the month preceding the baseline survey was less than $2400 (i.e., less than $28,800 per year). Missing data for each variable is indicated in Table 1. Across all waves of data collection, the largest share of participants in each demographic category was found to be: women; people born in Canada; not married nor living with a partner; with no dependents; and who had less than a college degree.

**Table 1:** Sample Demographic Characteristics by Data Collection Wave

	Wave 1 ***N*** = 401	Wave 2 ***N*** = 320	Wave 3 ***N*** = 311	Wave 4 ***N*** = 271
**Age (years)**				
Mean (SD)	43.9 (13.4)	44.5 (12.9)	44.3 (13.5)	44.3 (13.2)
**Gender**				
Men	163 (40.6%)	128 (31.9%)	116 (28.9%)	104 (25.9%)
Women	204 (50.9%)	169 (42.1%)	170 (42.4%)	145 (36.2%)
Gender diverse	34 (8.5%)	23 (5.7%)	25 (6.2%)	22 (5.5%)
Missing	0 (0%)	81 (20.2%)	90 (22.4%)	130 (32.4%)
**Education**				
Some college or less	248 (61.8%)	198 (49.4%)	192 (47.9%)	160 (39.9%)
College degree	67 (16.7%)	55 (13.7%)	52 (13.0%)	44 (11.0%)
Bachelor’s or graduate degree	51 (12.7%)	44 (11.0%)	38 (9.5%)	43 (10.7%)
Other	35 (8.7%)	23 (5.7%)	29 (7.2%)	24 (6.0%)
Missing	0 (0%)	81 (20.2%)	90 (22.4%)	130 (32.4%)
**Monthly Income (CAN$)**				
0–599	60 (15.0%)	36 (9.0%)	32 (8.0%)	21 (5.2%)
600–1199	163 (40.6%)	131 (32.7%)	112 (27.9%)	100 (24.9%)
1200–1799	75 (18.7%)	59 (14.7%)	75 (18.7%)	67 (16.7%)
1800–2399	22 (5.5%)	24 (6.0%)	31 (7.7%)	25 (6.2%)
2400 or more	12 (3.0%)	12 (3.0%)	31 (7.7%)	23 (5.7%)
Missing	69 (17.2%)	139 (34.7%)	120 (29.9%)	165 (41.1%)
**Born in Canada**				
Yes	276 (68.8%)	225 (56.1%)	211 (52.6%)	192 (47.9%)
No	101 (25.2%)	82 (20.4%)	80 (20.0%)	63 (15.7%)
Missing	24 (6.0%)	94 (23.4%)	110 (27.4%)	146 (36.4%)
**Ethnicity**				
White	214 (53.4%)	173 (43.1%)	163 (40.6%)	150 (37.4%)
First Nations/Metis/Inuit	36 (9.0%)	32 (8.0%)	25 (6.2%)	21 (5.2%)
Visible minority	151 (37.7%)	115 (28.7%)	123 (30.7%)	100 (24.9%)
Missing	0 (0%)	81 (20.2%)	90 (22.4%)	130 (32.4%)
**Marital status**				
Not married^a^	258 (64.3%)	220 (54.9%)	220 (54.9%)	193 (48.1%)
Married^a^	120 (29.9%)	89 (22.2%)	90 (22.4%)	78 (19.5%)
Missing	23 (5.7%)	92 (22.9%)	91 (22.7%)	130 (32.4%)
**Dependents**				
No dependent	209 (52.1%)	169 (42.1%)	168 (41.9%)	140 (34.9%)
One or more dependents	166 (41.4%)	137 (34.2%)	141 (35.2%)	129 (32.2%)
Missing	26 (6.5%)	95 (23.7%)	92 (22.9%)	132 (32.9%)
**Physical health (SF12 subscale)**				
Mean (SD)	45.2 (9.76)	43.9 (11.6)	44.2 (12.1)	43.5 (11.2)
**Mental health (SF12 subscale)**				
Mean (SD)	40.2 (11.3)	40.4 (11.7)	40.8 (13.9)	41.6 (11.9)

*Notes.* ‘Missing’ values include non-responses due to both attrition and unanswered questions within surveys. ^a^‘Married’ includes living with a partner

#### Food security

**Fig. 1:**
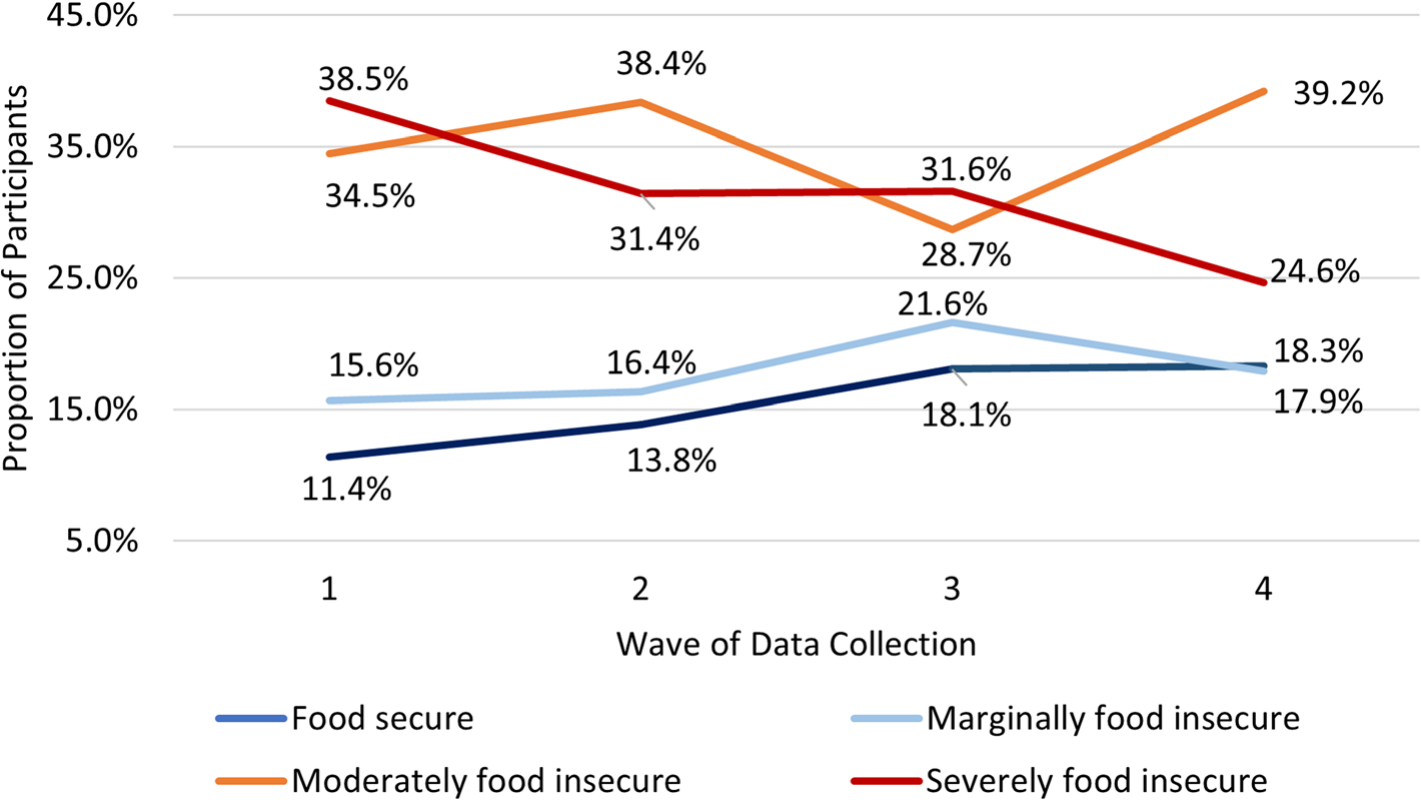
Proportion of Participants in Each Wave by Food Security Level

As show in Fig. 1 below, when comparing the overall change in food security from the first wave of data collection to the last wave, the proportion of people who were food *secure* increased, and the proportion of people that were *severely* food *insecure* decreased. Over the eighteen-month time span, there was an increase of seven percentage points (from 11 to 18%) in the proportion of participants in the food secure category, an increase of five percentage points (from 34 to 39%) in the moderately food insecure category, whereas there was an overall decrease of 14 percentage points (from 39 to 25%) in the severely food insecure category.

#### Frequency of food Bank use in the previous 3 months

Overall, the percentage of people who visited food banks three or more times in the preceding 3 months decreased over time. In the first wave of data collection, 52.1% of people who used the food banks used them three or more times in the previous 3 months, compared to 50.5% in wave 2, 42.4% in wave 3, and 27.440.6% in wave 4.

In the first wave of data collection, the majority of participants (52.1%) used food banks three or more times in the preceding 3 months, followed by those who visited the food banks once (23.2%) or twice (20.4%). The largest proportion of participants visited the food banks three or more times in all waves of data collection, compared to the proportions of participants that made either one or two visits in the preceding 3 months.

#### Perceived physical and mental health

The mean perceived physical health scores ranged from 45.2 (SD 9.76) in wave 1 to 43.5 (SD 11.2) in wave 4, while the mean perceived mental health scores ranged from 40.2 (SD 11.3) in wave 1 to 41.6 (SD 11.9) in wave 4 (Table 1).

No significant difference between the mean perceived physical and mental health by waves of data collection were detected, with the exception of a slight increase of 1.4 in the mean perceived mental health score between wave 1 and wave 4 (*p* < 0.001).

#### Descriptive statistics by levels of food security

Table 2 summarizes the demographic characteristics of participants over the four waves of data collection for each food security category. As shown in the table, participants who accessed the food banks were between the ages of 18 and 80 years old. There was an age gradient in food security: the mean age at baseline of people who were severely food insecure (42.2 years, SD 12.0) was 5 years lower than those who were food secure (47.2 years, SD 14.9). Across all four waves of data collection, there were 688 responses from women and 511 from men. Across food insecurity categories, the largest difference between men (35.4%) and women (57.6%) was in the moderately food insecure category.

**Table 2:** Demographic Characteristics by Level of Food Security (Using Aggregated Responses from All Four Waves of Data Collection)

	Food secure (***N*** = 192)	Marginally food insecure (***N*** = 226)	Moderately food insecure (***N*** = 446)	Severely food insecure (***N*** = 409)
**Age (years)**				
Mean (SD)	47.2 (14.9)	46.1 (14.0)	43.9 (13.0)	42.2 (12.0)
**Gender**				
Men	72 (37.5%)	99 (43.8%)	158 (35.4%)	173 (42.3%)
Women	112 (58.3%)	106 (46.9%)	257 (57.6%)	208 (50.9%)
Gender diverse	8 (4.2%)	21 (9.3%)	31 (7.0%)	28 (6.8%)
Missing	0 (0%)	0 (0%)	0 (0%)	0 (0%)
**Education**				
Some college degree or less	118 (61.5%)	141 (62.4%)	257 (57.6%)	274 (67.0%)
College degree	26 (13.5%)	29 (12.8%)	101 (22.6%)	61 (14.9%)
Bachelor’s or graduate degree	36 (18.8%)	32 (14.2%)	56 (12.6%)	47 (11.5%)
Other	12 (6.2%)	24 (10.6%)	32 (7.2%)	27 (6.6%)
Missing	0 (0%)	0 (0%)	0 (0%)	0 (0%)
**Household income (CAN$)**				
0–599	9 (4.7%)	21 (9.3%)	50 (11.2%)	66 (16.1%)
600–1199	51 (26.6%)	81 (35.8%)	183 (41.0%)	187 (45.7%)
1200–1799	46 (24.0%)	56 (24.8%)	100 (22.4%)	72 (17.6%)
1800–2399	23 (12.0%)	18 (8.0%)	37 (8.3%)	22 (5.4%)
2400 or more	21 (10.9%)	16 (7.1%)	20 (4.5%)	21 (5.1%)
Missing	42 (21.9%)	34 (15.0%)	56 (12.6%)	41 (10.0%)
**Born in Canada**				
Yes	108 (56.2%)	139 (61.5%)	330 (74.0%)	321 (78.5%)
No	77 (40.1%)	69 (30.5%)	95 (21.3%)	77 (18.8%)
Missing	7 (3.6%)	18 (8.0%)	21 (4.7%)	11 (2.7%)
**Ethnicity**				
White	99 (51.6%)	109 (48.2%)	251 (56.3%)	236 (57.7%)
First Nations/Metis/ Inuit	12 (6.2%)	15 (6.6%)	41 (9.2%)	46 (11.2%)
Visible minority	81 (42.2%)	102 (45.1%)	154 (34.5%)	127 (31.1%)
Missing	0 (0%)	0 (0%)	0 (0%)	0 (0%)
**Marital status**				
Not married^a^	116 (60.4%)	146 (64.6%)	323 (72.4%)	298 (72.9%)
Married^a^	74 (38.5%)	74 (32.7%)	115 (25.8%)	107 (26.2%)
Missing	2 (1.0%)	6 (2.7%)	8 (1.8%)	4 (1.0%)
**Dependents**				
No dependent	93 (48.4%)	120 (53.1%)	234 (52.5%)	232 (56.7%)
one or more dependents	96 (50.0%)	98 (43.4%)	201 (45.1%)	171 (41.8%)
Missing	3 (1.6%)	8 (3.5%)	11 (2.5%)	6 (1.5%)
**Physical health (SF12 subscale)**				
Mean (SD)	47.2 (10.7)	45.7 (11.2)	43.7 (11.3)	42.5 (10.7)
**Mental health (SF12 subscale)**				
Mean (SD)	48.8 (11.5)	44.5 (12.2)	39.6 (11.4)	35.8 (10.8)
**Frequency of use of food bank**^b^				
0	34 (17.7%)	30 (13.3%)	39 (8.7%)	39 (9.5%)
1	36 (18.8%)	55 (24.3%)	101 (22.6%)	90 (22.0%)
2	39 (20.3%)	38 (16.8%)	79 (17.7%)	86 (21.0%)
3 or more	83 (43.2%)	103 (45.6%)	225 (50.4%)	193 (47.2%)
Missing	0 (0%)	0 (0%)	2 (0.4%)	1 (0.2%)

*Notes.* ‘Missing’ values refer to unanswered questions within surveys. ^a^‘Married’ includes living with a partner. ^b^‘Frequency of use of food bank’ refers to the previous 3 months

Overall, out of 1111 responses on household income, 931 responses (83.8%) indicated an income of CAN$1799 or less per month. As well, an income gradient was found between people in different food security categories: among participants who were severely food insecure, only 5.1% had a monthly household income of CAN$2400 or more, compared to 10.9% of participants who were food secure.

There was a significant relationship between food security level and average perceived physical and mental health: those with higher levels of food security had higher levels of perceived health (Table 2). The mean physical health scores ranged from 47.2 for those who were food secure, to 42.5 for those who were severely food insecure. Similarly, the mean mental health scores ranged from 48.8 to 35.8 for the same categories.

### Contingency analysis

The Chi-squared test between CRC and Choice model was not significant (*p*-value = 0.7), which indicates that the variables are correlated. The same finding (*p*-value = 0.63) was found between the CRC and additional programming models, indicating that these variables are also correlated. As a result, we did not put the three variables in one model to predict food security scores, but instead tested each variable separately.

### Longitudinal regression models

We modeled the trajectory of the food security index, a continuous variable from one to ten where one is the most food secure, and ten is the most insecure. The results are summarized in Table 3.

**Table 3:** Food Security Trajectories by Food Bank Type: Integrated Within CRC, Offering a Choice Model, and Offering Additional Programming

Predictors of Food Insecurity (on HFSSM score)	**CRC**		**Choice model**		**Additional programming**	
	**Coef.**	**95% CI**	**Coef.**	**95% CI**	**Coef.**	**95% CI**
CRC (ref. not in CRC)	−.595**	[−.993, −.196]	–	–	–	–
Choice (ref. hamper model)	–	–	−.534**	[−.894, −.174]	–	–
Additional programming (ref. no additional programming)	–	–	–	–	−.164	[−.518, .191]
Age at baseline	−.028**	[−.042, −.013]	−.028**	[−.042, −.013]	−.029**	[−.044, −.015]
Gender (ref. men)						
Women	−.384*	[−.755, −.014]	−.395*	[−.765, −.024]	−.397*	[−.7731, −.021]
Gender diverse	.468	[−.555, 1.491]	.474	[−.550, 1.498]	.596	[−.441, 1.633]
Ethnicity (ref. First Nations)						
White	.391	[−.218, .999]	.357	[−.252, .966]	.376	[−.240, .992]
Visible minority	.185	[−.311, .681]	.159	[−.336, .654]	.139	[−.363, .641]
Born in Canada (ref. yes)						
No	−.572*	[−1.107, −.037]	−.537*	[− 1.070, −.003]	−.512	[− 1.052, .0279]
Income (ref. $0–$599)						
$600–$1799	−.151	[−.490, .189]	−.143	[−.483, .197]	−.154	[−.495, .188]
$1800 +	−.425	[−.864, .0138]	−.411	[−.850, .0278]	−.439	[−.880, .001]
Dependents (ref. yes)						
No	0.185	[−.153, .5222]	.218	[−.120, .555]	.193	[−.146, .532]
Marital status^a^ (ref. married)						
Not married^a^	−.243	[−.585, .099]	−.249	[−.591, .094]	−.224	[−.568, .120]
Physical health (PCS)	−.038**	[−.050, −.026]	−.037**	[−.049, −.0248]	−.037**	[−.049, −.0249]
Mental health (MCS)	−.051**	[−.061, −.040]	−.050**	[−.061, −.0394]	−.050**	[−.061, −.039]
Food bank use (ref. Accessed yes)						
No	.550**	[.163, .937]	.549**	[.158, .932]	.541**	[.152, .929]
Time (ref. baseline)						
6 months	−.788**	[−1.216, −.359]	−.780**	[− 1.208, −.351]	−.774**	[− 1.203, −.345]
12 months	−.989**	[−1.396, −.583]	−.987**	[− 1.393, −.581]	−.979**	[−1.386, −.571]
18 months	− 1.090**	[− 1.498, −.681]	−1.088**	[− 1.496, −.680]	−1.080**	[− 1.488, −.671]
Constant	11.17**	[10.02, 12.32]	11.13**	[9.98, 12.29]	11.10**	[9.93, 12.27]
Random-effects Parameters	**Estimate**	**95% CI**	**Estimate**	**95% CI**	**Estimate**	**95% CI**
SD (_cons)	1.439	[1.300, 1.592]	1.441	[1.303, 1.594]	1.462	[1.323, 1.617]
SD (Residual)	1.367	[1.296, 1.443]	1.367	[1.295, 1.442]	1.366	[1.295, 1.442]
Intraclass correlation	**0.53**	**[0.46–0.58]**	**0.56**	**[0.5–0.62]**	**0.51**	**[0.44–0.58]**

*Notes.* * indicates *p* < 0.05. ** indicates *p* < 0.01. ^a^‘Married’ includes living with a partner

The mixed effect regression model (a growth curve model/trajectory model) revealed that with every year increase in age at baseline, the food security score decreased by 0.03 units (i.e., food insecurity decreased with age). Being a woman was related to a decrease of 0.38 units in the food insecurity score compared to being a man. Being not born in Canada was related to 0.57 units decrease in the food insecurity score. Increased income was related to a decrease in food insecurity: having a monthly income of $1800 or more was related to 0.42 units of decreased food insecurity. Every 10 points increase in the physical health index was related to 0.4 units in decreased food insecurity; similarly, every 10 points increase in mental health index, was related to 0.5 units in decreased food insecurity.

After the first wave of data collection, food insecurity decreased over time by 0.78 units in wave 2, 0.98 units in wave 3, and 1.09 units in wave 4 (all compared to baseline), as shown in Table 3.

For participants who went to a food bank connected with a CRC, the food insecurity score was lower by 0.59 units compared to those who went to a regular food bank. For participants who went to a choice-model food bank, the food insecurity score was 0.53 units less than for those who went to hamper-model food banks. Additional onsite programming was not associated with any decrease or increase in food security. Having not accessed a food bank in the preceding 3 months was related to a higher likelihood of being food insecure, with the greatest increase observed for those who were marginally food insecure.

Having a higher age at baseline, being not born in Canada, married or living with a partner, with higher income, and higher perceived physical and mental health scores were associated with less food insecurity. For all other variables, the impact of the variable on the different food insecurity categories was not statistically significant. In the CRC model, the Intraclass correlation (rho) shows that 53% of the variance was explained by between-participants variance, as opposed to 56% in the Choice model, and 51% in the Program model.

## Discussion

In this study, 271 out of 401 participants (67.6%) responded during the final eighteen-month follow-up. Part of the observed attrition could be explained by findings from a large-scale longitudinal study conducted in Vancouver, Canada [52]. These researchers found that the majority of people who access food banks could be characterized as “short-term, transitional users who visited food banks a handful of times and disengaged after a few weeks or months of use,” and that the 9% who accessed food banks over a long-term accounted for 65% of all food bank visits. Thus, a significant number of the participants in our study at baseline may have only needed food assistance over a short term. We were often unable to contact participants for follow-ups because the contact information they provided was no longer valid (e.g., telephone was out of service and mailing address had changed), so it is impossible to say what changed in their life circumstances and whether they still had a need for food assistance.

As described above in the Methods section, those who participated in the 6-month follow-up were given a $5 grocery store gift card, and for the subsequent follow-ups the amount was increased to $10 to encourage retention due to the 20% attrition seen at 6 months. The increased incentive appears to have been successful since the incremental attrition rates at the 12- and 18-month time points were lower at 2% and 10%, respectively.

In terms of income, which is necessary for purchasing food, the results fit with what we would expect to find, as participants with the lowest income were more heavily represented in the severely food insecure category. Conversely, participants with CAD$1800 or more in monthly income were more heavily represented in the food secure category.

Food insecurity was higher for participants who were not married and not living with a partner. This may be because people who are married or live with a partner share major expenses like rent, and therefore may have more money for food if they both have incomes. As well, if one partner loses some or all of their income, the other partner’s income may ‘cushion’ the economic impact. Lastly, single parents working in the service industry find it problematic to work varied hours for relatively low wages, and also schedule paid childcare, so they may not be able to earn sufficient income to maintain their food security [53].

In terms of gender, the majority of participants in our study were women (683 total responses in all four waves by women compared to 502 responses by men). The greatest disparity was in the moderately food insecure category, in which there were 38.5% fewer responses from men than from women. Our regression analysis found that food insecurity among women in our study was 0.38 points lower on the 10-point food insecurity scale (where a lower score means less food insecurity).

The higher proportion of women participants in our study may have been due to an unintended gender bias in the recruitment process, or the results above (lower number of men, but with higher food insecurity than women) may also reflect sociocultural attitudes that men should behave stoically and not ask for help except in dire circumstances. A 2012 study in Montréal, Canada involved in-depth interviews with 22 men experiencing poverty, followed by six discussion groups to validate the results, which suggested that “asking for help can be diametrically opposed to traditional masculine roles” and that, when facing a serious problem, men will ask for help only as a last resort [54].

There were notable differences between the demographics of the participants in this study and those of the general population of Ottawa, based on the 2016 Census figures from Statistics Canada. In terms of education, the census showed that 63.7% of people in Ottawa had a postsecondary certificate, diploma, or degree [55], compared to 29.4% of the participants in the baseline survey. Our result closely matches that of a 2005 study in Toronto, Canada, which found that 27.4% of people accessing food banks in Toronto had completed college or university [56]; however, our result is very different from a US study using national data which found that less than 8% of people that received assistance from food pantries between 2002 and 2014 had a college degree (US meaning, similar to university) [57]. The Toronto study found a drastic increase – from 12% in 1995 to 53% in 2005 – in the percentage of immigrants with some college or university education among immigrants who received assistance from food banks, so the higher numbers of educated people accessing food banks in Canada, versus the United States, may reflect Canadian immigration policy.

We found that participants born in Canada reported significantly higher food insecurity than those who were not born in Canada. This may also be due to Canadian immigration policies, which require people coming to Canada as immigrants to be skilled or well educated or to possess a prescribed amount of liquid assets [58].

In terms of income, only 3% of the participants at baseline reported a monthly household income of $2400 ($28,800 per year) or more, compared to 86% of all residents in the city of Ottawa having an annual household income of $30,000 or more in 2016 [55]. Although 17% of the participants in our study did not provide income information, the results still indicate a huge income gap between people who visit food banks and other people in Ottawa.

In terms of ethnicity, 9% of the participants in our study were Indigenous (First nations, Metis, or Inuit), which is almost double the 4.6% of people in all of Ottawa who are Indigenous [55]. This result echoes the urgent need to address the inequity in food security faced by off-reserve Indigenous people in Canada [59].

Consistent with previous research that found poorer health was correlated with food insecurity [3–5, 7], we found the mean perceived physical and mental health scores to be below the general population mean of 50 points [46] for all of our participants. Moreover, perceived physical and mental health scores both showed gradients across food insecurity levels, such that health scores decreased as the severity of food insecurity increased. Participants in the food secure category scored closest to 50 points, with means of 47.2 for perceived physical health and 48.8 for perceived mental health, suggesting that their health was close to that of the general population.

While previous research has also found evidence of gradients in mental and physical health according to the severity of food insecurity [60–65], those studies depended on national health surveys (i.e., the Canadian Community Health Survey, and the National Health and Nutrition Examination Survey in the U.S.) to obtain data on household food insecurity and did not focus specifically on people who access food banks. Other studies have found that less than one quarter of food insecure households in Canada relied on food banks, and that the people who do access food banks were not a representative subset of the food insecure population, having substantially lower incomes and higher rates of receiving social assistance benefits than food insecure people who had not accessed food banks [38, 39]. As such, the examination of perceived physical and mental health in the current study relates to a unique subset of the food insecure population. Our finding that the largest proportions of participants across all waves were in the CAD$600–1199 bracket may reflect that many of the participants in our study received modest social assistance benefits as their source of income.

Physical health scores ranged from 47.2 for food secure participants to 42.5 for those who were severely food insecure. Mental health scores were even lower for moderately and severely food insecure participants at 39.6 and 35.8, respectively. Since the standard deviation (SD) of the SF-12 health scores is 10 points, obtaining mean results that are more than one SD below the average of 50 points is concerning. In comparison, another study [66] with a similar sample size of food insecure adults (*n* = 325) drawn from a population survey in the Lower Mississippi Delta in the United States, obtained mean physical and mental health scores of 45.7 and 46.5, respectively, using the SF-12 scales. The mean physical health score falls within the 47.2–42.5 range obtained in the current study; however, the mean mental health scores that we obtained were much lower (35.8–39.6, versus 46.5 in the US study), so this difference suggests poorer overall mental health for people who rely on food banks, compared to food insecure people in the general population. This is in consonance with previously cited research [38, 39], which reported that people who access food banks are not a representative subset of all people who report being food insecure. It is also important to note that the physical health scores did not differ significantly between the four waves of data collection, and that the mental health scores showed a statistically significant, albeit slight improvement.

In this study, we didn’t analyse the associations between different food banking models and physical and mental health; however, due to the increasing prevalence of food banks using novel approaches to providing food assistance, we believe that future research to examine possible associations with health is certainly warranted.

The longitudinal reduction in food insecurity that we observed with food banks integrated in a Community Resource Centre is consistent with the findings of our colleague Enns [44] at the 6-month time point. The initial reduction in the mean food insecurity score was the most pronounced: 0.79 points out of 10 after 6 months, compared to a decrease of 0.99 points at 12 months and 1.09 points at 18 months (all compared to baseline). Although the consecutive decreases in the food insecurity scores seem to indicate further improvements at 12 and 18 months, the differences were not statistically significant, so larger studies would be needed to confirm if, in fact, there is a continued reduction in food security over time for those who access CRC-type food banks. In any case, the overall reduction in food insecurity that we observed for people who access CRC-type food banks is encouraging because they are also able to access the health and social services offered by CRCs when they visit the Centre for food assistance.

We also found a small but significant difference in food security according to the food distribution model of the food bank. Across all four waves of data collection, the proportions of participants were lower in the moderately and severely food insecure categories if they accessed food banks using the Choice model, compared to participants who visited food banks offering food hampers. Our regression analysis also showed that when food banks used the Choice model, longitudinal food insecurity was 0.53 less (on the 10-point scale) compared to food banks that used the hamper approach. This adds to the findings of the six-month follow-up by Enns [44], who reported a significant increase in fruit and vegetable consumption by people who accessed food banks that employed a Choice model of food distribution. The Choice model may be especially beneficial for those who must avoid certain foods for medical reasons (e.g., lactose intolerance, low sugar diets for diabetics, gluten allergy) or for cultural/religious reasons (e.g., avoiding processed foods that contain animal-based ingredients such as gelatin and broth, which are not considered kosher or halal). Studies have also shown that people prefer to choose food items that they need (based on personal or cultural preferences or dietary requirements) and not have to throw away food they dislike or cannot use if they receive a pre-packed box [67, 68]. The benefit of the choice approach may therefore be threefold: lower observed levels of food insecurity when the Choice model is offered, lower levels of waste, and conferring more dignity on the consumer. However, one drawback of the Choice model perceived by people who accessed choice food pantries was longer line-ups [67].

Finally, we believe it is important to consider that the food security level measured in this study is the self-reported level of participants *while* accessing food banks (whereas most of the reviewed literature provides food insecurity data primarily from people who do not rely on food banks). We found that 63.5% of participants who described themselves as food *secure* reported that they had visited a food bank two or more times in the previous 3 months (Table 2); since food banks provide only a few days’ worth of food, it appears that low levels of food insecurity may be temporarily eased by food banks. On the other hand, a more disconcerting observation is that 47.2% of participants in the severely food insecure category reported this level even after visiting a food bank three or more times in the previous 3 months. Similarly, 50.4% of participants in the moderately food insecure category reported that level after also visiting food banks three or more times in the previous 3 months. From these results we can see that food banks may temporarily alleviate food insecurity for some people, whereas many others remain moderately or severely food insecure.

Because household food insecurity is, by definition, due to financial constraints, our findings lend support to the need for public policy changes, such as increases in social support payments or implementing a guaranteed basic income, which several other studies have proposed [69–72]. In Canada and other high-income countries, food insecure people with insufficient incomes currently have to rely on a bureaucratic, costly, and stigmatizing ‘patchwork’ of social assistance programs administered by different levels of government; because of the shortcomings of existing social safety nets, many researchers have advocated specifically for a simplified guaranteed basic income as a more effective solution [73–76].

### Limitations

There are several possible limitations to the findings of this study. First, since the analysis was restricted to one Canadian city with a high median household income – $86,451 per year in Ottawa in 2016, versus $70,336 across all of Canada [55] – it may not be representative of other physiographic regions in Canada or other countries.

Furthermore, participation was restricted to a convenience sample of English and French speaking adults; thus, some members of the population may be inadequately represented in the sample. Researchers approached participants to take part in the survey; as a result, there may have been bias due to self-selection of volunteers. Recall, acquiescence response and social desirability biases are all known to influence survey respondents [77, 78]. Moreover, the data was collected several times over pre-established observation points in this longitudinal study; hence, we cannot account for circumstances occurring in between those time periods. Finally, although the present study analysed a diverse group of food banking models, it lacked a comparable sample, specifically one that was food insecure but did not access food banks. Without a control group, we cannot be sure that the results were not due to other factors (i.e., unobserved or unmeasured covariates).

### Strengths

This study addresses a gap in the evaluation of contemporary food assistance programs by providing current data on the associations between food insecurity and food banking approaches. This study also adds important evidence on the compromised physical and mental health of food insecure people who rely on food banks for assistance. The key strength of this study is that it helps to fill these gaps by providing longitudinal data, collected over 18 months, on patterns of food insecurity over time, and modelling the impact of different food bank approaches on food insecurity scores.

## Conclusion

We found significant reductions in food insecurity for people who accessed food banks that offered a Choice model of food distribution and food banks that were integrated within Community Resource Centres. Although our results show a small improvement in food security overall, it is important to note that generally, most participants still reported moderate or severe food insecurity at the end of the 18-month study, indicating a clear need for an effective long-term solution such as a guaranteed income to provide financial stability for people facing food insecurity in Canada. One positive finding was that the mean perceived mental health score was slightly higher at the 18-month point compared to baseline, possibly due to the small improvement in food security. Since our results found poor self-reported health among the subset of food insecure people who access food banks, additional larger and longitudinal studies that explore and address the unique health concerns of this population are vitally needed.

## Data Availability

The datasets used and/or analysed during the current study are available from the corresponding author on reasonable request.
