# Correction to: The impact of novel and traditional food bank approaches on food insecurity: a longitudinal study in Ottawa, Canada

**DOI:** 10.1186/s12889-021-10981-9

**Published:** 2021-05-19

**Authors:** Anita Rizvi, Rania Wasfi, Aganeta Enns, Elizabeth Kristjansson

**Affiliations:** 1 School of Psychology, Faculty of Social Sciences, University of Ottawa, 136 Jean-Jacques Lussier Pvt, Room VNR5015, Vanier Hall, Ottawa, Ontario K1N 6N5 Canada; 2 Centre for Surveillance and Applied Research, Public Health Agency of Canada, Government of Canada, Ottawa, Canada

**Correction to: BMC Public Health 21, 771 (2021)**

**http://orcid.org/10.1186/s12889-021-10841-6**

It was highlighted that the original article [1] contained an error in the Results section, subheading Descriptive statistics/ *Frequency of food Bank use in the previous 3 months* in the last sentence of the first paragraph. The sentence erroneously read “ … compared to 50.5% in wave 2, 42.4% in wave 3, and 27.440.6% in wave 4.” Instead of “ … compared to 50.5% in wave 2, 42.4% in wave 3, and 40.6% in wave 4”. The original article has been updated.
